# Bibliometric analysis of scientific publications on waterpipe (narghile, shisha, hookah) tobacco smoking during the period 2003-2012

**DOI:** 10.1186/1617-9625-12-7

**Published:** 2014-04-13

**Authors:** Sa’ed H Zyoud, Samah W Al-Jabi, Waleed M Sweileh

**Affiliations:** 1 Poison Control and Drug Information Center (PCDIC), College of Medicine and Health Sciences, An-Najah National University, Nablus, Palestine; 2 Department of Pharmacology and Toxicology, College of Medicine and Health Sciences, An-Najah National University, Nablus, Palestine; 3 WHO Collaborating Centre for Drug Information, National Poison Centre, Universiti Sains Malaysia (USM), Penang, Malaysia; 4 Department of Clinical and Community Pharmacy, College of Medicine and Health Sciences, An-Najah National University, Nablus, Palestine

**Keywords:** Bibliometric, Waterpipe tobacco smoking, Narghile, Shisha, Hookah, Scopus

## Abstract

**Background:**

Waterpipe tobacco smoking has spread worldwide. However, the evaluation of scientific output in the field of waterpipe tobacco smoking has not been studied yet. The main objectives of this study were to analyze worldwide research output in the waterpipe tobacco smoking field, and to examine the authorship pattern and the citations retrieved from the Scopus database for over a decade.

**Methods:**

Data from January 1, 2003 through December 31, 2012 were searched for documents with specific words regarding waterpipe tobacco smoking as “keywords” in the title. Scientific output was evaluated based on a methodology developed and used in other bibliometric studies: (a) total and trends of contributions in waterpipe tobacco smoking research between 2003 and 2012; (b) authorship patterns and research productivity; (c) collaboration patterns; (d) the citations received by the publications; and (e) areas of interest of the published papers.

**Results:**

Worldwide there were 334 publications that met the criteria during the study period. The largest number of publications in waterpipe tobacco smoking were from the United States of America (USA) (33.5%), followed by Lebanon (15.3%), and France (10.5%). The total number of citations at the time of data analysis (October 18, 2013) was 4,352, with an average of 13 citations per document and a median (interquartile range) of 4.0 (1.0–16.0). The *h*-index of the retrieved documents was 34. The highest *h*-index by country was 27 for the USA, followed by 20 for Syrian Arab Republic and Lebanon.

**Conclusions:**

The present data reveal a promising rise and a good start for research activity in the field of waterpipe tobacco smoking. More effort is needed to bridge the gap in waterpipe smoking-based research and to promote better evaluation of waterpipe smoking, risks, health effects, or control services worldwide.

## Background

Tobacco use is the most important single risk factor for both heart diseases and cancer and is also the most preventable leading cause of morbidity and mortality, annually contributing to around 6 million deaths worldwide, and is estimated to exceed 8 million deaths by 2030 [1]. Unfortunately, scientific research tends to focus on tobacco use methods that are widespread in developed countries (e.g., cigarettes, tobacco cessation) and often does not consider methods prevalent in developing countries, such as the waterpipe [2,3]. The waterpipe, known in many cultures under different names (e.g., narghile, shisha, hookah), is a centuries-old tobacco-use method that has traditionally been associated with Middle Eastern societies. In fact, waterpipes are now commonplace in Arab cultures, and are served in many cafes and restaurants in the Eastern Mediterranean Region (EMR). In the waterpipe, inhalation of charcoal-heated air is done through perforated aluminium foil separating the charcoal from the flavoured tobacco to become smoke; this smoke is sweetened and flavoured (for example, apple, watermelon, mint) [3,4]. Other forms of tobacco may contain less sweeteners/flavours and are called Ajami, or Tumba’k. Smoke travels down the body of pipe, and bubbles through water in the bowl to cool on its way to smokers’ lungs [3,5]. The emergence of waterpipe smoking as a global risk to public health is evidenced by the promising increase in the past decade of studies devoted to the waterpipe [2,4,6-10].

Tobacco smoking is on the rise and as a multi-disciplinary field of study, has resulted in growing research that takes into account almost all the regions that have experienced the greatest increases in bioscience and healthcare science production [11,12]. In contrast, the evolution of the overall output of scientific research in the field of tobacco use has been poorly explored to date and there are few internationally published reports on research activity regarding tobacco use [13-17].

The evaluation of scientific research in a certain field is an essential task where the purpose of evaluation is to determine, and where possible to improve, productivity. Scientific progress is one of the most important indicators of community and economic development of different countries [18]. Bibliometric analysis is a useful tool to obtain a clear picture of the current state of scientific research in particular areas and allocates researchers to recognise and undertake new lines of research [19]. This type of analysis is a type of research method used in library and information sciences. It utilises quantitative analysis and statistics to obtain the bibliographical works within a given field, topic, institute, or country [20,21]. The most important key indicators of research capacity and productivity in the field of tobacco include the number of peer-reviewed scientific journal articles, numbers of total citations, and type of publications [16,22].

The objectives of this study were to analyze worldwide research output in the waterpipe tobacco smoking field, and the citations retrieved from the Scopus database between 2012-2013.

## Methods

### Search strategy

This study relied on data extracted from Scopus that was published between January 1, 2003 and December 31, 2012. The choice of the study duration was based on the assumption that the last decade represents a better picture of the pattern of publications and citations received in a certain field when using bibliometric methods [23-26]. A comprehensive online search was performed using SciVerse, Scopus, which is one of the world’s largest databases of peer-reviewed literature. Scopus covers nearly 18,000 titles from 5,000 publishers worldwide, and contains 41 million records and provides 100% MEDLINE coverage [27].

Waterpipe terminology can depend upon region, and includes names such as “shisha” (Egypt, Saudi Arabia), “narghile” “nargile,” or “arghile” (Israel, Jordan, Lebanon, Syria), and “hookah” or “hubble bubble” (Africa and India) [3]. The keywords entered into the Scopus engine to achieve the objectives of this study were selected from the related studies on waterpipe usage [2,3,28,29]. All the following selected “keywords” were entered as “Article Title”: “waterpipe,” “sheesha,” “hookah,” “hukka,” “narghile,” “shisha,” “hubble bubble,” “hubbly-bubbly,” “qalyan,” “ghelyan,” “nargile,” “nargila,” “water pipe,” and “water-pipe.” Most of the time the term “water pipe” is used as a pipe for conveying water. Then, to avoid this confusion, the following keywords: “smoking,” “tobacco,” “smoke,” “smoker,” “nicotine,” smokeless,” non-smoking,” and “secondhand” were entered as “all fields.” All subject areas for a 10-year period (2003-2012) were selected for this research: health sciences, social sciences, life sciences, and physical sciences. We excluded documents that published as errata or as a chapter in a book. We also excluded those documents in which the primary focus was not a dimension of waterpipe tobacco smoking.

The collected data were used to generate the following information: (a) total and trends of contributions in waterpipe tobacco smoking research between 2003 and 2012; (b) authorship patterns and research productivity; (c) collaboration patterns; (d) the citations received by the publications; and (e) areas of interest of the published papers. All searches were completed within one day on October 18, 2013 to avoid bias due to the daily update of databases.

### Ethical approval

The Institutional Review Board (IRB) at An-Najah National University does not require submission of an IRB application for such study. Previous similar publications by the same group of authors were officially waived from submission of an IRB application [30]. The IRB considered that there is no risk for human subjects in such publications since the data are based on published literature rather than human subjects and did not involve any interactions with human subjects.

### Statistical analysis

Data from Scopus were exported to Microsoft Office Excel® and then transferred to the Statistical Package for Social Sciences (SPSS; SPSS Inc., Chicago, IL, USA) program version 15 for analysis. Variables that are not normally distributed are expressed as median (Q1–Q3: interquartile range) and categorical data are expressed as numbers with percentages. The *h*-index for the data collected from Scopus is presented. The *h*-index represents the number of citations received for each of the documents in descending order, while the h-graph measures the impact of a set of documents and displays the number of citations per document. Impact factors (IF) for the journals were evaluated using the Journal Citation Report (JCR; Web of Knowledge) 2012 science edition by Thomson Reuters (New York, NY, USA).

## Results

The total number of documents published globally about waterpipe tobacco smoking from 1976 until 2012 was 353 while that between 2003 – 2012 was 334. This means that 94.6% of the total global research productivity in waterpipe tobacco smoking was published during the specified study period. Analysis in this manuscript was based on the 334 documents since it represents approximately 95% of the global research in the field of waterpipe smoking in the past 3 decades. The 334 documents consisted of 219 (65.6%) original journal articles, 53 (15.9%) letters to the editor, 33 (9.9%) review articles, and 29 (8.7%) other types of publications, with an average of 33 documents per year. The annual number of documents published in the past decade (2003–2012) indicate that waterpipe tobacco smoking research productivity during the past decade was low in the first few years but showed an obvious increase in recent years (Table 1).

**Table 1:** Total article included in bibliometric analysis in the field of waterpipe tobacco smoking by publication year

**Year**	**Total N** **=** **334 (%)**
2003	6 (1.8)
2004	14 (4.2)
2005	10 (3.0)
2006	11 (3.3)
2007	24 (7.2)
2008	49 (14.7)
2009	40 (12.0)
2010	53 (15.9)
2011	59 (17.7)
2012	68 (20.4)

The retrieved documents were published in 162 peer-reviewed journals. Table 2 shows the 10 top journals in which waterpipe tobacco smoking related articles were published. Nineteen documents (5.7%) were published in *Nicotine and Tobacco Research* whereas 11 (3.3%) were published in *Tobacco Control*, 11 (3.3%) were published in *Food and Chemical Toxicology*, and 9 (2.7%) were published in *BMC Public Health*.

**Table 2:** Ranking top 10 journals in which waterpipe tobacco smoking related articles were published based on impact factors

**SCR**^**a**^	**Journal**	**Frequency**	IF (2012)*
1^st^	*Nicotine and Tobacco Research*	19 (5.7)	2.477
2^nd^	*Tobacco Control*	11 (3.3)	4.111
3^rd^	*Food and Chemical Toxicology*	11 (3.3)	3.010
4^th^	*BMC Public Health*	9 (2.7)	2.076
5^th^	*International Journal of Tuberculosis and Lung Disease*	8 (2.4)	2.610
6^th^	*Addictive Behaviors*	6 (1.8)	2.021
6^th^	*Journal of the Pakistan Medical Association*	6 (1.8)	0.409
6^th^	*Revue Des Maladies Respiratoires*	6 (1.8)	0.495
9^th^	*Asian Pacific Journal of Cancer Prevention*	5 (1.5)	1.271
9^th^	*American Journal of Public Health*	5 (1.5)	3.930

*Abbreviations*: *SCR* Standard Competition Ranking, *IF* impact factor.

^a^Equal journals have the same ranking number, and then a gap is left in the ranking numbers.

*The impact factor was reported according to Institute for Scientific Information (*ISI*) journal citation reports (JCR) 2012.

All retrieved documents were published from 42 countries. Table 3 shows a list of rankings of the 10 countries whose researchers published the largest number of articles in the field of waterpipe tobacco smoking during the period between 2003 and 2012. When the data were analyzed by country, the largest number of publications in the field of waterpipe tobacco smoking was from the United States of America (USA); (33.5%), followed by Lebanon (15.3%), and France (10.5%); (Table 3). The total number of citations at the time of data analysis (October 18, 2013) was 4,352, with an average of 13 citations per document and a median (interquartile range) of 4.0 (1.0–16.0). The highest median (interquartile range) number of citations was 30 (6–55) for Syrian Arab Republic (SAR), followed by 11 (3–35) for Lebanon. The *h*-index of the retrieved documents was 34 (i.e., 34 documents had been cited at least 34 times at the time of data analysis). The highest *h*-index was 27 for the USA, followed by 20 for SAR and Lebanon. Furthermore, the highest number of collaborations with international authors for each country was held by the USA, with 58 documents, followed by 31 documents for the SAR and 24 documents for Lebanon (Table 3).

**Table 3:** The top 10 ranking of the most productive countries that published the largest number of articles in the field of waterpipe tobacco smoking during the period from 2003 to 2012

**SCR**^**a**^	**Countries**	**Articles (%)**	**H-index**	**Median citation (Q1-Q3)**	**Average citation**	**Collaborations with foreign countries**	**Total documents from collaboration**
1^st^	USA	112 (33.5)	27	8 (2-26)	19.7	20	58
2^nd^	Lebanon	51 (15.3)	20	11 (3-35)	27.3	7	24
3^rd^	France	35 (10.5)	7	2 (0.0-5.2)	5.7	7	9
4^th^	SAR	31 (9.3)	20	30 (6-55)	37	8	31
5^th^	Germany	22 (6.6)	11	9 (0.0-50)	27.6	3	9
6^th^	Canada	21 (6.3)	7	4 (0.5-11)	8.2	11	16
7^th^	Iran	18 (5.4)	5	2.5 (0.0-7)	3.6	5	5
8^th^	Jordan	16 (4.8)	7	3 (2-15)	9	6	9
9^th^	Egypt	13 (3.9)	5	3 (1-17)	8.3	4	9
9^th^	Pakistan	13 (3.9)	6	7 (0.0-18.8)	9.8	4	4
	Others (32)						

*Abbreviations*: *SCR* Standard Competition Ranking, *USA* United States of America, *SAR* Syrian Arab Republic, Q1-Q3 lower quartile – upper quartile.

^a^Equal countries have the same ranking number, and then a gap is left in the ranking numbers.

Table 4 presents the areas of interest of the scientific articles. Medicine was the most researched topic, represented by 268 (80.2%) articles. The second most researched topic was pharmacology, toxicology and pharmaceutics by 38 (11.4%) followed by biochemistry, genetics and molecular biology for 27 (8.1%) articles and environmental science for 23 (6.9%). Table 5 shows the top 10 most productive institutions in the field of waterpipe tobacco smoking. The most productive institution was *American University of Beirut*, Lebanon (13.2% of total publications), followed by *Virginia Commonwealth University*, USA (9.6%), and *Syrian Center for Tobacco Studies*, SAR (8.7%).

**Table 4:** Ranking the top 10 areas of interest of the published papers worldwide in the field of waterpipe tobacco smoking during the study period

**SCR**	**Areas of interest**	n (%)*
1^st^	Medicine	268 (80.2)
2^nd^	Pharmacology, Toxicology and Pharmaceutics	38 (11.4)
3^rd^	Biochemistry, Genetics and Molecular Biology	27 (8.1)
4^th^	Environmental Science	23 (6.9)
5^th^	Social Sciences	18 (5.4)
6^th^	Agricultural and Biological Sciences	13 (3.9)
6^th^	Psychology	13 (3.9)
8^th^	Dentistry	11 (3.3)
9^th^	Nursing	10 (3.0)
10^th^	Neuroscience	4 (1.2)

*Abbreviation*: *SCR* Standard Competition Ranking.

*total exceeds 100% as data are overlapping due to multidiscipline interaction.

**Table 5:** Ranking of the top 10 highly productive institutions in the field of waterpipe tobacco smoking during the study period

**SCR**^**a**^	**Institutions**	**No. of documents (%)**
1^st^	American University of Beirut, Lebanon	44 (13.2)
2^nd^	Virginia Commonwealth University, USA	32 (9.6)
3^rd^	Syrian Center for Tobacco Studies, SAR	29 (8.7)
4^th^	University of Memphis, USA	25 (7.5)
5^th^	Universite Paris-Sud XI, France	22 (6.6)
6^th^	Westfälische Wilhelms-Universität Münster, Germany	10 (3.0)
7^th^	Jordan University of Science and Technology, Jordan	9 (2.7)
8^th^	University of Balamand, Lebanon	8 (2.4)
9^th^	University of Florida, USA	7 (2.1)
10^th^	Wayne State University, USA	6 (1.8)
10^th^	Bundesinstitut für Risikobewertung, Germany	6 (1.8)

*Abbreviations*: *SCR* Standard Competition Ranking, *USA* United States of America, *SAR* Syrian Arab Republic.

^a^Equal institutions have the same ranking number, and then a gap is left in the ranking numbers.

## Discussion

Reducing waterpipe smoking-caused morbidity and mortality worldwide requires an understanding of how various countries have progressed in waterpipe tobacco smoking scientific research. Such an understanding is instrumental for the development of an effective plan to respond to this progress and garner public and political support for it [31-33]. This study was limited to 334 documents extracted from Scopus, bearing article titles with terms related to waterpipe tobacco smoking and, therefore, cannot be generalized to the waterpipe tobacco smoking literature covered by other databases such as Google Scholar. Although the number of citations for each publication might differ from one search engine to another and hence the final results would differ by database selected, the Scopus search engine remains one of the best available databases for analyzing and tracking citations and comparing citations to different research groups and different institutions [11]. A study that compared Scopus, Google Scholar, PubMed, and Web of Knowledge found that PubMed is considered an important resource for clinicians and researchers, while Scopus offers the capability for citation analysis and covers a wider journal range [11,34-36].

The total number of documents related to tobacco obtained by entering only the words “tobacco or smoking” in Scopus search engine as an article title without specifying the name of any country was 36,407 documents. This number is limited to these terms and represents the total global research productivity in tobacco or smoking during the past decade (unpublished data). Only 334 (0.91% of the total global research productivity in tobacco) documents in the field of waterpipe tobacco smoking were retrieved. These findings indicate that waterpipe tobacco smoking has not been extensively studied as cigarette smoking.

Although waterpipe use is spreading and millions of current waterpipe smokers are found across the world, there has been surprisingly little research addressing tobacco smoking using a waterpipe [37]. All these findings support the need to conduct more research on waterpipes, the issues related to their use, and to distribute the information on their health risks to all countries.

In the present study, bibliometric indicators were used to describe worldwide research output in the waterpipe tobacco smoking field during the last decade. Based on the authors’ knowledge, this is the first article to analyze the quantity of waterpipe smoking-based research from around the world. Research indicators showed that research activity in this field was neglected and was only restricted to 42 countries. This paper also adds to the emerging bibliometric literature within tobacco research. The total publications found in Scopus between 2003 and 2012 showed a yearly increase. Waterpipe tobacco smoking research productivity has followed the general explosion in scientific productivity observed in the last decade and especially in recent years [14,16,17]. Our study showed that there were some countries, such as the USA, Lebanon and France, where the total waterpipe tobacco smoking research productivity during this 10-year period was clearly higher than in the other countries. Therefore, it would have been more interesting to know how the growth of tobacco research in these countries differed in quality rather than in quantity, as shown by the *h*-index. It was observed that countries such as the USA, Lebanon, and SAR demonstrate high *h*-index values. The number of articles with international collaborations from these countries was high.

We reported that contributions from the outside of the USA appeared to increase steadily during the period of study. Specifically, the current data indicate that Lebanon, SAR, Jordan, Egypt and Iran have been the major research contributors from the Middle East. The top ten countries in the field of waterpipe tobacco smoking publication productivity are different from familiar nations for other scientific productivity ranking [38].

To the best of our knowledge, this study is the first of its kind to obtain initial data regarding worldwide publication productivity in the waterpipe tobacco smoking field from the Scopus database, a database that is used to evaluate the performance of institutes and their members. This study is not without limitations, most of which are the same as those of studies performed in other biomedical fields [30,39]. First of all, we used Scopus criteria for including waterpipe-related keywords in our study. Articles published in non-Scopus-cited journals were not included, although they would likely contribute to scientific productivity. Another limitation is that some articles did not point out waterpipe and related terms in article titles; however, these terms were mentioned throughout the text. Therefore, it is possible that the number of publications analyzed in this study did not exactly represent all waterpipe smoking-based research activity. Finally, it should be noted that research output for certain institutions could have been under-estimated because of writing their English names differently in different articles. Therefore, such institutions might have 2 or more author profiles in Scopus because their names were written differently in different articles.

## Conclusion

The present data reveal a promising rise and a good start for research activity in the field of waterpipe tobacco smoking. More effort is needed to bridge the gap in waterpipe smoking-based research and to promote better evaluation of waterpipe smoking, risks, health effects, or control services around the world.

## Abbreviations

SPSS: Statistical Package for Social Sciences; SAR: Syrian Arab Republic; ISI: Institute for Scientific Information; EMR: Eastern Mediterranean Region; SCR: Standard Competition Ranking; USA: United States of America; JCR: Journal Citation Report; IFs: Impact factors; IRB: Institutional Review Board; Q1-Q3: lower quartile – upper quartile; WHO FCTC: World Health Organization Framework Convention on Tobacco Control.

## Competing interests

The authors declare that they have no competing interests.

## Authors’ contributions

All authors were involved in drafting the article, and all authors approved the final version to be submitted for publication. SZ conceived of the study conception and design, organized and supervised the data collection, and provided analysis, interpretation, and writing. SA and WS participated in the study design, and provided critical revision of manuscript for important intellectual content.

## References

[B1] World Health Organization WHO report on the global tobacco epidemic, 2011: warning about the dangers of tobacco 2011 [cited 2013 17, October]; Available from: http://www.who.int/tobacco/global_report/2011/en/

[B2] Maziak W The global epidemic of waterpipe smoking Addict Behav 2011 36 1 5 10.1016/j.addbeh.2010.08.030 20888700 PMC4135081

[B3] Maziak W Ward KD Afifi Soweid RA Eissenberg T Tobacco smoking using a waterpipe: a re-emerging strain in a global epidemic Tob Control 2004 13 327 333 10.1136/tc.2004.008169 15564614 PMC1747964

[B4] Maziak W The waterpipe: an emerging global risk for cancer Cancer Epidemiol 2013 37 1 4 10.1016/j.canep.2012.10.013 23196170 PMC4143981

[B5] Maziak W The Waterpipe: A New Global Threat to CV Health Global Heart 2012 7 179 181 10.1016/j.gheart.2012.03.003 25691314

[B6] Cobb C Ward KD Maziak W Shihadeh AL Eissenberg T Waterpipe tobacco smoking: an emerging health crisis in the United States Am J Health Behav 2010 34 275 285 10.5993/AJHB.34.3.3 20001185 PMC3215592

[B7] Hammal F Mock J Ward KD Eissenberg T Maziak W A pleasure among friends: how narghile (waterpipe) smoking differs from cigarette smoking in Syria Tob Control 2008 17 e3 10.1136/tc.2007.020529 18375726

[B8] Harrabi I Maaloul JM Gaha R Kebaili R Maziak W Ghannem H Comparison of cigarette and waterpipe smoking among pupils in the urban area of Sousse, Tunisia Tunis Med 2010 88 470 473 20582881

[B9] Maziak W The waterpipe: time for action Addiction 2008 103 1763 1767 10.1111/j.1360-0443.2008.02327.x 18778388 PMC2588474

[B10] Maziak W Nakkash R Bahelah R Husseini A Fanous N Eissenberg T Tobacco in the Arab world: old and new epidemics amidst policy paralysis Health Policy Plan 2013 Article in press 10.1093/heapol/czt055 PMC4153301 23958628

[B11] de Granda-Orive JI Alonso-Arroyo A Roig-Vazquez F Which data base should we use for our literature analysis? Web of Science versus SCOPUS Arch Bronconeumol 2011 47 213 21281995 10.1016/j.arbres.2010.10.007

[B12] De Granda-Orive JI Villanueva-Serrano S Aleixandre-Benavent R Valderrama-Zurián JC Alonso-Arroyo A García Río F Jiménez Ruiz CA Solano Reina S González Alcaide G Network of international scientific collaboration on smoking: analysis of coauthorship through the Science Citation Index (1999-2003) Gac Sanit 2009 23 222.e234-e243 19464767 10.1016/j.gaceta.2008.05.002

[B13] Garcia-Lopez JA Bibliometric analysis of Spanish scientific publications on tobacco use during the period 1970-1996 Eur J Epidemiol 1999 15 23 28 10.1023/A:1007564809921 10098992

[B14] Cohen JE Chaiton MO Planinac LC Taking stock a bibliometric analysis of the focus of tobacco research from the 1980s to the 2000s Am J Prev Med 2010 39 352 356 10.1016/j.amepre.2010.06.003 20837286

[B15] Warner KE Tam J Koltun SM Growth in Tobacco Control publications by authors from low- and middle-income countries Tob Control 2013 Article in press 10.1136/tobaccocontrol-2012-050762 23291400

[B16] Kira A Glover M Bullen C Viehbeck S Publications as an indicator of increased tobacco control research productivity (quantity and quality) in New Zealand Nicotine Tob Res 2011 13 474 478 10.1093/ntr/ntr029 21436296

[B17] Nykiforuk CI Osler GE Viehbeck S The evolution of smoke-free spaces policy literature: a bibliometric analysis Health Policy 2010 97 1 7 10.1016/j.healthpol.2010.03.001 20381188

[B18] Lin JY Rosenblatt D Shifting patterns of economic growth and rethinking development J Econ Policy Reform 2012 15 171 194 10.1080/17487870.2012.700565

[B19] De Battisti F Salini S Robust analysis of bibliometric data JISS 2013 22 269 283

[B20] Wallin JA Bibliometric methods: pitfalls and possibilities Basic Clin Pharmacol Toxicol 2005 97 261 275 10.1111/j.1742-7843.2005.pto_139.x 16236137

[B21] Tan J Fu H-Z Ho Y-S A bibliometric analysis of research on proteomics in Science Citation Index Expanded Scientometrics 2014 98 1473 1490 10.1007/s11192-013-1125-2

[B22] McDonald PW Viehbeck S Robinson SJ Leatherdale ST Nykiforuk CI Jolin MA Building research capacity for evidence-informed tobacco control in Canada: a case description Tob Induc Dis 2009 5 12 10.1186/1617-9625-5-12 19664224 PMC2746799

[B23] Zyoud SH Al-Jabi SW Sweileh WM Worldwide research productivity of paracetamol (acetaminophen) poisoning: a bibliometric analysis (2003-2012) Hum Exp Toxicol 2014 Article in press 10.1177/0960327114531993 24758786

[B24] Zyoud SH Al-Jabi SW Sweileh WM Awang R A bibliometric analysis of toxicology research productivity in Middle Eastern Arab countries during a 10-year period (2003-2012) Health Res Policy Syst 2014 12 4 10.1186/1478-4505-12-4 24443999 PMC3899442

[B25] Zyoud SH Al-Jabi SW Sweileh WM Awang R A bibliometric analysis of research productivity of Malaysian publications in leading toxicology journals during a 10-year period (2003–2012) Hum Exp Toxicol 2014 Article in press 10.1177/0960327113514101 24505047

[B26] Zyoud SH Al-Jabi SW Sweileh WM Awang R Assessing the scientific research productivity of a leading toxicology journal: A case study of Human & Experimental Toxicology from 2003 to 2012 SAGE Open Med 2014 2 2050312114523424 10.1177/2050312114523424 PMC4607183 26770709

[B27] Scopus SciVerse Scopus fact sheet. SciVerse® Scopus 2013 [cited 2013 14, September]; Available from: http://www.elsevier.com/online-tools/scopus

[B28] Akl EA Gaddam S Gunukula SK Honeine R Jaoude PA Irani J The effects of waterpipe tobacco smoking on health outcomes: a systematic review Int J Epidemiol 2010 39 834 857 10.1093/ije/dyq002 20207606

[B29] De Leon E Smith KC Cohen JE Dependence measures for non-cigarette tobacco products within the context of the global epidemic: a systematic review Tob Control 2013 Article in press 10.1136/tobaccocontrol-2012-050641 23783510

[B30] Sweileh WM Zyoud SH Sawalha AF Abu-Taha A Hussein A Al-Jabi SW Medical and biomedical research productivity from Palestine, 2002–2011 BMC Res Notes 2013 6 41 10.1186/1756-0500-6-41 23375070 PMC3566958

[B31] Nakkash R Afifi R Maziak W Research and activism for tobacco control in the Arab world Lancet 2014 383 392 393 10.1016/S0140-6736(13)62381-8 24452050

[B32] World Health Organization WHO report on the global tobacco epidemic, 2013: enforcing bans on tobacco advertising, promotion and sponsorship: executive summary 2013 [cited 2014 10, April]; Available from: https://extranet.who.int/iris/restricted/bitstream/10665/85381/1/WHO_NMH_PND_13.2_eng.pdf

[B33] World Health Organization WHO Framework Convention on Tobacco Control 2003 [cited 2013 11, October]; Available from: http://whqlibdoc.who.int/publications/2003/9241591013.pdf

[B34] Falagas ME Pitsouni EI Malietzis GA Pappas G Comparison of PubMed, Scopus, web of science, and Google scholar: strengths and weaknesses FASEB J 2008 22 338 342 17884971 10.1096/fj.07-9492LSF

[B35] Kulkarni AV Aziz B Shams I Busse JW Comparisons of citations in Web of Science, Scopus, and Google Scholar for articles published in general medical journals J Am Med Assoc 2009 302 1092 1096 10.1001/jama.2009.1307 19738094

[B36] Tadmouri GO Bissar-Tadmouri N A major pitfall in the search strategy on PubMed Saudi Med J 2004 25 7 10 14758370

[B37] World Health Organization (WHO) Waterpipe tobacco smoking: health effects, research needs and recommended actions by regulators 2005 Geneva, Switzerland: World Health Organization [cited 2013 19, October]; Available from: http://www.who.int/tobacco/global_interaction/tobreg/Waterpipe%20recommendation_Final.pdf

[B38] Essential Science Indicators Top 20 Countries in ALL FIELDS, 2001-August 31, 2011 2012 [cited 2013 20, September]; Available from: http://archive.sciencewatch.com/dr/cou/2011/11decALL/

[B39] Zyoud SH Al-Jabi SW Sweileh WM A Scopus-based examination of tobacco use publications in Middle Eastern Arab countries during the period 2003-2012 Harm Reduct J 2014 11 Article in Press 10.1186/1477-7517-11-14 PMC4012166 24885706

